# Third International Biannual Evolution and Cancer Conference (Evolutionary Trade‐offs and Clinical Consequences) Meeting report. San Francisco, CA, USA. 10–13 December 2015

**DOI:** 10.1111/eva.12359

**Published:** 2016-03-04

**Authors:** Benjamin Roche, Frédéric Thomas

**Affiliations:** ^1^ Centre for Ecological and Evolutionary Research on CancerMontpellierFrance; ^2^ UMI IRD/UPMC 209 UMMISCOParisFrance; ^3^ UMR CNRS/IRD/UM 5290 MIVEGECMontpellierFrance

**Keywords:** cancer, ecology, evolution, trade‐offs

## Introduction

Despite recent notable progress in medical sciences, cancer remains a leading cause of human death worldwide, accounting for about one‐quarter of human deaths in wealthy countries and about one‐eighth worldwide (Ferlay et al. 2010). In fact, the progress in cancer research has been slower compared with that achieved related to other pathologies, such as cardiovascular disorders. This is mainly because of the enormous complexity of this disease, which exhibits sophisticated cellular mechanisms that are the targets of evolutionary processes driven by random genetic and epigenetic mutations.

Indeed, cancer development and progression represent evolutionary and ecological processes acted on by Darwinian selection, which drives cancer cells along evolutionary landscapes and culminates in resistance to immune attack, malignant progression, metastasis, and sometimes even contagion. It thus appears crucial to adopt this evolutionary perspective for our understanding of cancer, its origin, the possible ways to control neoplastic progression, and to prevent therapeutic failures (Merlo et al. 2006; Aktipis and Nesse 2013; Thomas et al. 2013).

Although these ideas originated in the mid‐seventies, many promising opportunities for the application of evolutionary biology to carcinogenesis and oncology remain unexplored and questions unanswered. For example, to what extent do the evolutionary sciences offer relevant tools for understanding and predicting cancer progression, and are they more applicable to certain malignancies than others? Can we alter the competition between cancerous and noncancerous cells? What are the selective effects of our therapies? Why and how do animal species differ in their predisposition to cancer? These relevant questions and many others were the focus of presentations and discussions at the Third Biannual International Evolution and Cancer Conference that was held from 10 to 13 December 2015 in San Francisco (CA, USA). This conference brings clinicians and evolutionary scientists from all over the world (mostly the US, UK, and France) together every 2 years to present the latest advances in this growing field. There were nearly 75 attendees at the 2015 conference.

The theme of this year was ‘Evolutionary Tradeoffs and Clinical Consequences’. Trade‐offs are pervasive in biological systems, and the evolutionary dynamics underlying cancer are no exception. Conference presentations explored the ways in which trade‐offs have shaped the evolution of cancer suppression systems and the role of trade‐offs in the progression of tumors from benign to malignant. A consideration of evolutionary trade‐offs can also help us to identify challenges and opportunities in cancer therapies and new horizons for cancer prevention.

## Summary of presentations

The meeting started on 10 December with a keynote address by Susan Rosenberg (Baylor College of Medicine, Houston, TX, USA), who explained how bacteria and cancer cells regulate mutagenesis and their ability to evolve. Dr. Rosenberg presented the molecular mechanism of stress‐induced mutation in *Escherichia coli*; she pointed out the similarities with human cancer cells and suggested that stress‐control responses can regulate mutagenesis in time and genomic space. Such an approach may lead to treatments that focus on the evolutionary process rather than its product.

The rest of the meeting was organized around several plenary talks followed by sessions focused on the introduced topic. On 11 December, Hanna Kokko (University of Zurich, Switzerland) gave a plenary talk on the perspective of using cancer as a component of life‐history theory. She demonstrated the utility of understanding cancer more broadly in wild animals (especially Peto's paradox, which is the unexpected absence of a relationship between cancer incidence and animal body size, and therefore the number of cells) to ultimately improve prevention and/or treatment in humans. She also showed that cancer is generally neglected as a factor in life‐history evolution.

### Session ‘Organismal Evolution and Peto's Paradox’ (chaired by Josh Schiffman, University of Utah Health Care, Salt Lake City, UT, USA)

Speakers: Josh Schiffman, Leonard Nunney (University of California, Riverside, CA, USA), and Robert Noble (Institut des Sciences de l'Evolution, Montpellier, France).

This session mostly focused on the possible explanations of Peto's paradox and the relevance of the field of comparative oncology. These topics were addressed through examples at very different scales, from elephants and their numerous copies of p53 (which protect them more efficiently from cancer) to comparisons among human organs according to their size. These talks highlighted the complexity of this relationship because of the numerous trade‐offs involved and the specificity of each scale to respond to the emergence of cancerous cells, from animal species to different organ tissues.

The second plenary talk of the day was given by Sarah Hill (Texas Christian University, Fort Worth, TX, USA), who showed the link between life‐history trade‐offs and vulnerability to cancer. She illustrated how life‐history trade‐offs made in early life can increase cancer risk in adulthood, especially how growing up in resource‐poor environments promotes eating in the absence of real necessity (a pattern that promotes survivability in resource scarce environments), which can in turn increase cancer risk in environments that are food rich. Similarly, she showed that individuals with less reactive NK cells (which play a major role in the elimination of cancerous cells) are generally associated with a range of behavioral outcomes associated with faster life‐history strategies (e.g., younger age at reproduction, short life span), especially those promoting cancer.

### Session ‘Somatic Evolution and Cancer Stem Cells’ (chaired by Leonard Nunney, University of California, Riverside, CA, USA)

Speakers: James de Gregori (University of Colorado, Denver, Aurora, CO, USA CO, USA), Lucie Laplane (Centre National de la Recherche Scientifique, Paris), and Vincent Cannataro (University of Florida, Gainesville, FL, USA).

This session covered the most recent progress into the drivers of clonal evolution from the very different perspectives of a philosophical point of view to *in vivo* experimentation to *in silico* modeling. The role of aging, the causal relationship between cancer stem cells and clonal evolution, and the importance of considering clonal evolution in a small stem cell population were discussed. These talks highlighted progress made in the understanding of clonal evolutionary processes and also identified important gaps that need to be filled. The day ended with a public lecture by Barbara Natterson‐Horowitz (University of California, Los Angeles, CA, USA), who showed how veterinary science can transform human medicine through evolutionary biology.

The third day started with a plenary talk by Daniel Nettle (Newcastle University, UK), who showed how early life adversity affects cellular aging in the European starling (*Sturnus vulgaris*). Using an experimental system, he showed that early adversity, which could be nutritional and/or social, can accelerate telomere attrition, which in turn can be associated with altered adult behavior. Such results demonstrate how early life events may accelerate cellular aging and thus impact on the probability of cancer emergence.

### Parallel session ‘Tumor Heterogeneity’ (chaired by Carlo Maley, Arizona State University, Tempe, AZ, USA)

Speakers: Oana Carja (University of Pennsylvania, Philadelphia, PA, USA), Henry Heng (WayneState University, Detroit, MI, USA), Diego Mallo (University of Vigo, Spain), Diego Chowell (Arizona State University), Pierre Martinez (Barts Cancer Institute, UK) and Jeffrey Townsend (Yale School of Medicine, New Haven, CT, USA).

This session explored the many different ways to characterize the drivers of tumor heterogeneity, which is a core problem in chemotherapy resistance. The session then explored the different methodologies that can be used, from agent‐based modeling to the evolutionary analysis of longitudinal single‐cell clonal analysis. The importance of treatment in the dynamics of tumor heterogeneity was also discussed. It was shown that metastatic cancer does not necessarily require sophisticated tumor heterogeneity and that metastatic processes can begin quite early in the tumor construction process.

### Parallel session ‘Methods and Experimental Biology’ (chaired by Luca Ermini, Institute of Cancer Research, London, UK)

Speakers: Robert Beardmore, Carlos Reding, Ivana Gudelj (University of Exeter, UK); Luca Ermini and Mary Kuhner (University of Washington, Seattle, WA, USA).

This session mostly focused on the combination of experimental and theoretical studies that can determine the drivers of genetic diversity observed in cancer. Talks suggested than stable genetic polymorphisms can arise from trade‐offs in simple environments, but some trade‐off shapes can also underpin it. Other topics, such as an extension of the current r‐K selection theory to explain the complexity of cancerous cell resources or the hazards of considering only driver and passenger mutations, were also discussed.

The afternoon session began with a plenary talk by Joel Brown (University of Illinois, Chicago, and Moffitt Cancer Center, Tampa, FL, USA) on the mechanisms of coexistence in tumor heterogeneity for which he drew analogies with known ecological patterns in animal species communities. He emphasized the necessity for researchers working on cancer evolution to consider the ecological part of this evolution because tumors cannot be summarized only as a collection of mutant genotypes and phenotypes, but rather as some cell lineages that fill a finite number of distinct niches.

### Session ‘Evolutionary Approaches Relevant to Cancer Treatment’ (chaired by Amy Boddy, ArizonaState University, Tempe, AZ, USA)

Speakers: Mark Robertson‐Tessi, Amy Boddy, Chandler Gatenbee (Moffitt Cancer Research Center, Tampa, FL, USA); William Chang (Albert Einstein College of Medicine, New York, NY, USA).

The aim of this session was to show how evolutionary approaches could bring new solutions to the struggle against cancer. One solution proposed by Robertson‐Tessi solution was the possibility of combining different therapies targeting different aspects of clonal evolution (especially phenotypic plasticity) to avoid resistance to chemotherapy; this solution used a mathematical model calibrated with an experimental model. The importance of the spatial dimension was covered extensively, including the emergence of resistance and cellular strategies to escape attack from the immune system.

The day ended with a keynote address by Marco Gerlinger (The Institute of Cancer Research, London, UK) on the tools available to reconstruct and predict the evolution of cancerous cells. Using large‐scale and ultra‐sensitive genomic technologies, evolutionary trajectories of tumors can be described and quantified. Moreover, phenotype‐directed genetic analyses start to reveal the potential functional effects of newly acquired genotypes. This talk ended with some suggestions for future research that could help to predict the evolutionary dynamics within a tumor.

The last day started with a plenary talk given by Aurora Nedelcu (University of New Brunswick, Fredericton, NB, Canada) on the possibility to exploit trade‐offs to understand and expose cancer's evolutionary vulnerabilities. Using experimental evolutionary systems, she showed how we can identify the new trade‐offs that emerge in cancerous cells and what potential weak points could be exploited for future treatments.

### Session ‘Cancer on the Evolution of Life’ (chaired by Athena Aktipis, Arizona State University, Tempe, AZ, USA)

Speakers: Frédéric Thomas (Center for Ecological and Evolutionary Research on Cancer, Montpellier, France); Athena Aktipis, Jekaterina Erenpreisa (Latvian Biomedical Research and Study Centre, Latvia); Carlo Maley (Biodesign Institute, School of life Sciences, Arizona State University, Tempe AZ, USA).

This last session focused on the impact of cancer on the evolution of organisms. Using experimental and theoretical systems, different impacts of cancer on an organism's life‐history traits—from fecundity to life cycles through cellular competition and cancer susceptibility—were discussed. The last talk by Carlo Maley covered the main problems in cancer biology (namely risk stratification, prevention, early detection, therapeutic responses, and acquired therapeutic resistance) and discussed how evolutionary ecology has recently been applied to them.

A poster session was also organized that were presented by 15 teams of researchers from several countries around the world. A broad range of topics were addressed, including the role of immunity, the influence of vitamin C on cancer, and the different ways to connect clonal evolution to tumor heterogeneity. The award for the best poster was presented to Camille Jacqueline (Center for Ecological and Evolutionary Research on Cancer, Montpellier, France) for her work on the indirect role of infectious diseases on the proliferation of cancer cells through immune system trade‐offs.

## Creation of the international society for evolution, ecology and cancer

This conference was the first to be organized under the responsibility of the International Society for Evolution, Ecology and Cancer (ISEEC), which was created on this occasion. The mission of this scientific society is to advance cancer research and clinical management by employing evolutionary, comparative, and ecological approaches and principles to cancer biology, prevention, and treatment. By bringing together cancer biologists, evolutionary biologists, ecologists, quantitative modelers, bioinformaticians, and clinicians, the society seeks to enable collaboration at the interface of these fields and to promote the exchange of research findings, novel methodologies, and theoretical frameworks across disciplines. ISEEC supports education and outreach efforts to train the next generation of scientists in the evolution and ecology of cancer and also engages the public in fundamental questions about the nature and evolutionary origins of cancer.

This three‐day conference was the third one organized on the topic of evolution and cancer; the two previous conferences also took place in San Francisco (in 2011 and 2013). Even though the scientific community in this new discipline is not yet growing very rapidly, the variety of the topics addressed highlights the dynamism of this emerging scientific field. Moreover, vastly different perspectives have been examined—from the impact of cancer on the evolution of life to the impact of evolution on cancer. New exciting insights for both the evolutionary and medical communities can be expected in the near future.
